# Temporary loop end ileostomy reduces the risk of stoma outlet obstruction: a comparative clinical study in patients undergoing restorative proctocolectomy and ileal pouch-anal anastomosis

**DOI:** 10.1007/s00595-024-02944-5

**Published:** 2024-10-23

**Authors:** Takayuki Ogino, Yuki Sekido, Tsunekazu Mizushima, Makoto Fujii, Ryota Mori, Mitsunobu Takeda, Tsuyoshi Hata, Atsushi Hamabe, Norikatsu Miyoshi, Mamoru Uemura, Yuichiro Doki, Hidetoshi Eguchi

**Affiliations:** 1 Department of Gastroenterological Surgery, Graduate School of Medicine, Osaka University, Osaka, Japan; 2 Department of Gastroenterological Surgery, Osaka Police Hospital, Osaka, Japan; 3 Division of Health Sciences, Graduate School of Medicine, Osaka University, Osaka, Japan

**Keywords:** Ileal pouch-anal anastomosis, Small bowel obstruction, Stoma outlet obstruction, Restorative proctocolectomy, Ulcerative colitis

## Abstract

**Purpose:**

Stoma outlet obstruction (SOO) is a serious complication of restorative proctocolectomy (RPC) and ileal pouch-anal anastomosis (IPAA). When the ileal mesentery to the pouch is under excessive tension, the ileum near the ileostomy twists easily, causing SOO. Loop-end ileostomy (EI) for fecal diversion was introduced in 2021 to prevent SOO, and we aimed to verify whether temporary EI reduces the incidence of SOO in RPC and IPAA patients relative to loop ileostomy (LI).

**Methods:**

This study included 106 consecutive RPC and IPAA patients with a diverting ileostomy and categorized them into LI (*n* = 75) or EI (*n* = 31) groups. The clinical characteristics of the patients were analyzed and compared.

**Results:**

Patient characteristics were similar between the groups, except for higher preoperative steroid use in the LI group (38.7%; *p* = 0.0116). There were no significant differences between the groups in anatomical factors, such as abdominal wall thickness and the height-adjusted distance between the root of the superior mesenteric artery and the bottom of the external anal sphincter. There were no significant differences in surgery-related factors, with ≥ 90% of the patients in each group undergoing laparoscopic procedures. A multivariate logistic regression analysis revealed that EI significantly reduced the risk of SOO relative to LI (OR, 0.18; 95% CI 0.03–0.92; *p* = 0.0399).

**Conclusion:**

EI reduced SOO levels after RPC and IPAA and may be beneficial for cases in which anastomosis is challenging.

## Introduction

**Fig. 1:**
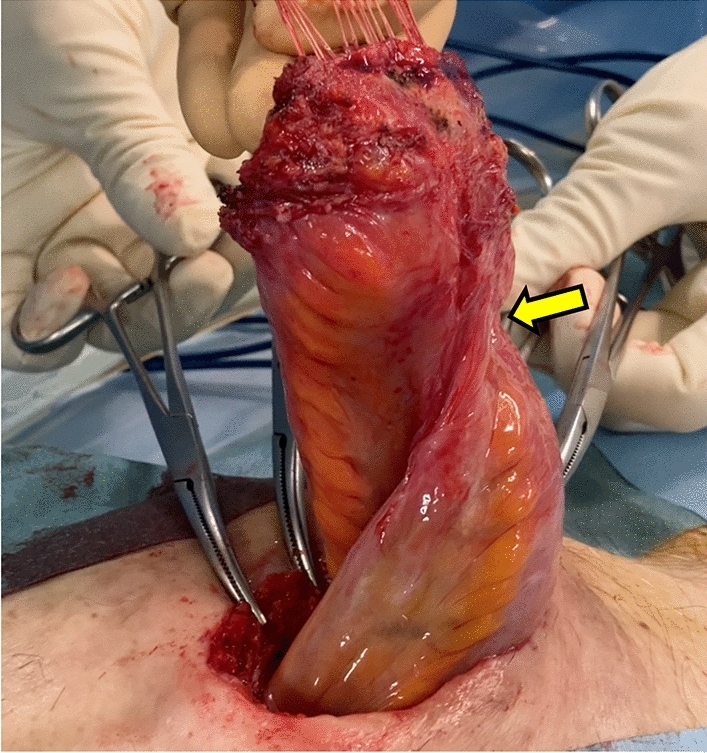
Stoma outlet obstruction (SOO) due to angulation of the ileum near the stoma site. The arrow indicates the obstruction site

For ulcerative colitis (UC) and familial adenomatous polyposis (FAP), restorative proctocolectomy (RPC) and ileal pouch-anal anastomosis (IPAA) are performed as standard surgical procedures. Temporary diverting ileostomy is often used to prevent pouch-related complications, including anastomotic leakage. Most surgeons prefer loop ileostomy (LI) for fecal diversion after RPC and IPAA because it is easier to perform stoma closure than loop end ileostomy (EI) [1]. However, temporary LI can be complicated by stoma-related small bowel obstruction (SBO), also called stoma outlet obstruction (SOO), due to the angulation of the ileum near the stoma site, which results in narrowing (Fig. 1).

Previous studies have shown that RPC and IPAA are associated with a higher incidence of postoperative complications related to ileostomy in comparison to other types of abdominal surgery [2]. In particular, SOO occurs with an incidence of 20–40% after RPC and IPAA, while the incidence after low anterior resection for rectal cancer is < 15% [3]. SOO not only delays the resumption of eating but also prolongs the length of hospital stay [4].

Our earlier study revealed that a longer distance between the root of the superior mesenteric artery and the bottom of the external anal sphincter (rSMA-bEAS) was a risk factor for SOO after RPC and IPAA [5]. In cases where the ileal mesentery to the pouch is under excessive tension, the ileum near the ileostomy can easily twist and recovery can be compromised. These findings suggest that surgeons should strive to reduce mesenteric tension as much as possible, not only for anastomosis but also for SOO. Although several studies have suggested techniques to resolve this situation, such as sufficient mobilization of the small intestine, division of the mesenteric serosa, and division of the blood vessels to stretch the mesentery [6–8], SOO remains a major problem after RPC and IPAA.

**Fig. 2:**
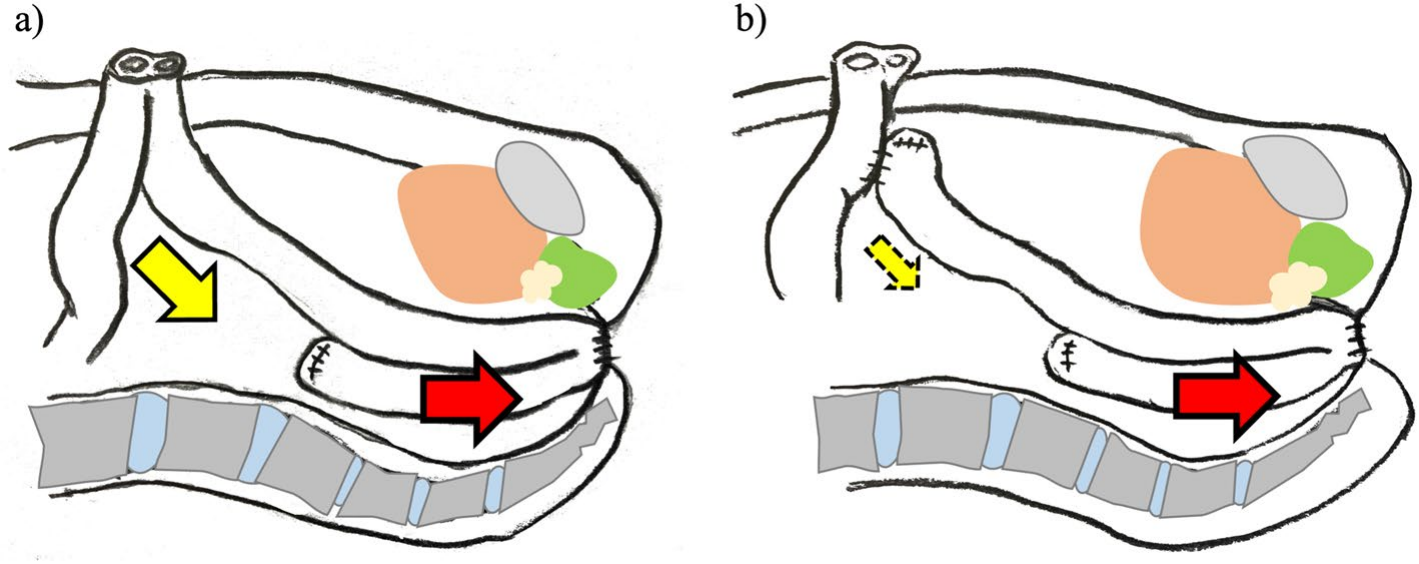
Tension of the ileal mesentery. The red arrow indicates the traction force from the ileal pouch-anal anastomosis. **a** Loop ileostomy causes cephalocaudal tension on the ileal mesentery. **b** This tension can be alleviated by loop end ileostomy

LI was originally performed after IPAA as a routine temporary fecal diversion procedure. We hypothesized that LI could cause cephalocaudal tension on the ileal mesentery, which might be alleviated by performing EI with partial division of the mesentery, including dissection of marginal vessels (Fig. 2). Therefore, in 2021, after considering the results of a previous study, we introduced EI to prevent SOO. Here, we conducted a comparative study to verify whether temporary EI reduces the incidence of SOO relative to LI in consecutive patients undergoing RPC and IPAA.

## Methods

### Patients

**Fig. 3:**
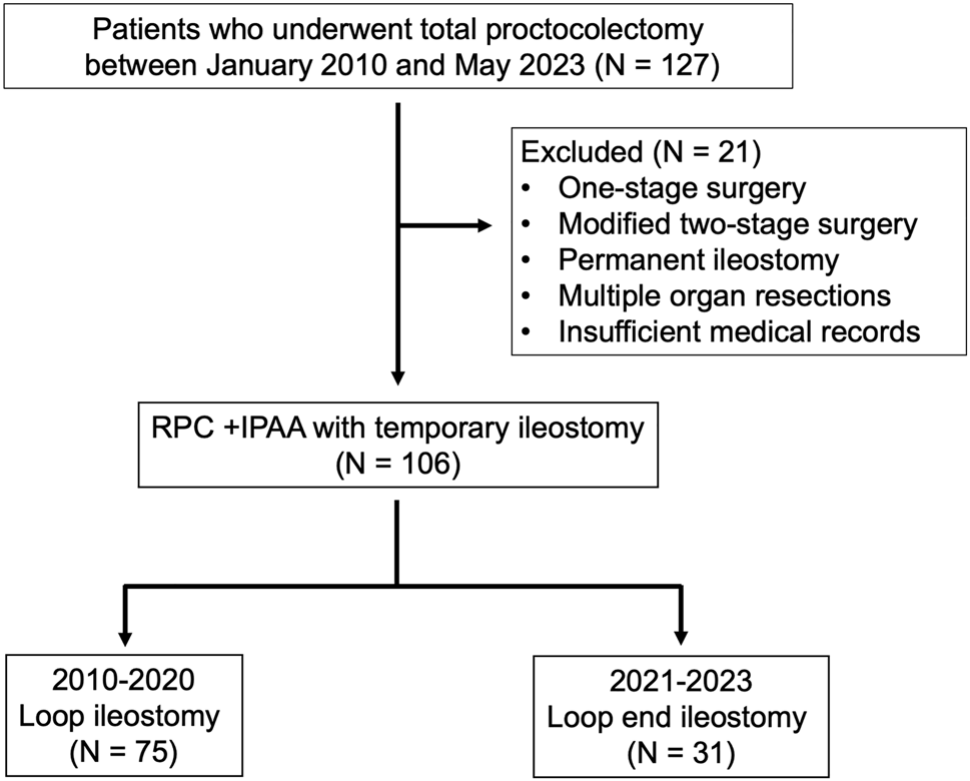
Flowchart of patient selection. *RPC* restorative proctocolectomy, *IPAA* ileal pouch-anal anastomosis

One hundred six consecutive patients who underwent RPC and IPAA with diverting ileostomies at Osaka University Hospital and Osaka Police Hospital between January 2010 and May 2023 were included in this study. Patients who underwent 1-stage surgery, modified 2-stage surgery (described below), permanent ileostomy, multiple organ resection, or whose medical records were insufficient were excluded. LI was performed as a method of fecal diversion until 2020. This was replaced by EI (with partial division of mesentery), with EI performed for all patients from January 2021 onwards. Patients were grouped by ileostomy (LI or EI), and the incidence of SOO, other complications, and patient characteristics were compared between the two groups (Fig. 3). The indications for surgery were determined at treatment team conferences attended by gastroenterologists, surgeons, radiologists, nutritionists, and nurses. All surgeries were performed by board-certified colorectal surgeons with well-established skills. The study protocol was approved by the Ethics Review Board of Osaka University Hospital (#15,028–3).

### Surgical procedure

RPC and IPAA for patients with UC could include up to three stages based on whether a diverting ileostomy was created and when closure was performed in accordance with the Diagnostic Criteria and Treatment Guidelines for Research on Intractable Inflammatory Bowel Diseases, supported by Health and Labor Sciences Research Grants from the Ministry of Health, Labor, and Welfare of Japan. One-stage surgery involved RPC and IPAA without ileostomy. Two-stage surgery involved RPC and IPAA with ileostomy, followed by stoma closure. Modified 2-stage surgery involved subtotal colectomy (STC) followed by residual rectal resection (RR) and IPAA without ileostomy. Three-stage surgery involved STC with ileostomy, RR and IPAA with ileostomy, and stoma closure. Two-stage surgery was selected for all patients with FAP. Stapled IPAA was performed for refractory or severe UC, whereas handsewn IPAA was performed for FAP and UC with cancer, dysplasia, or severe anal canal inflammation. Lymph node dissection was routinely performed for patients with cancer.

The standard procedure has been described previously [5]. Briefly, following sufficient mobilization of the right colon, an ileal J-pouch of 15 cm in length was created without dissection of the ileocecal artery. The pouch was confirmed 2 cm caudal to the lower border of the pubic bone. Mobilization of the left colon and rectum was performed to complete RPC. A diverting ileostomy was created on the oral side, 30–80 cm from the site of IPAA. The stoma site (umbilicus or right or left abdomen) was selected at the discretion of the surgeon. A lengthwise incision was made at the stoma site in the rectus muscle fascia, which was large enough to allow the passage of two fingers.

**Fig. 4:**
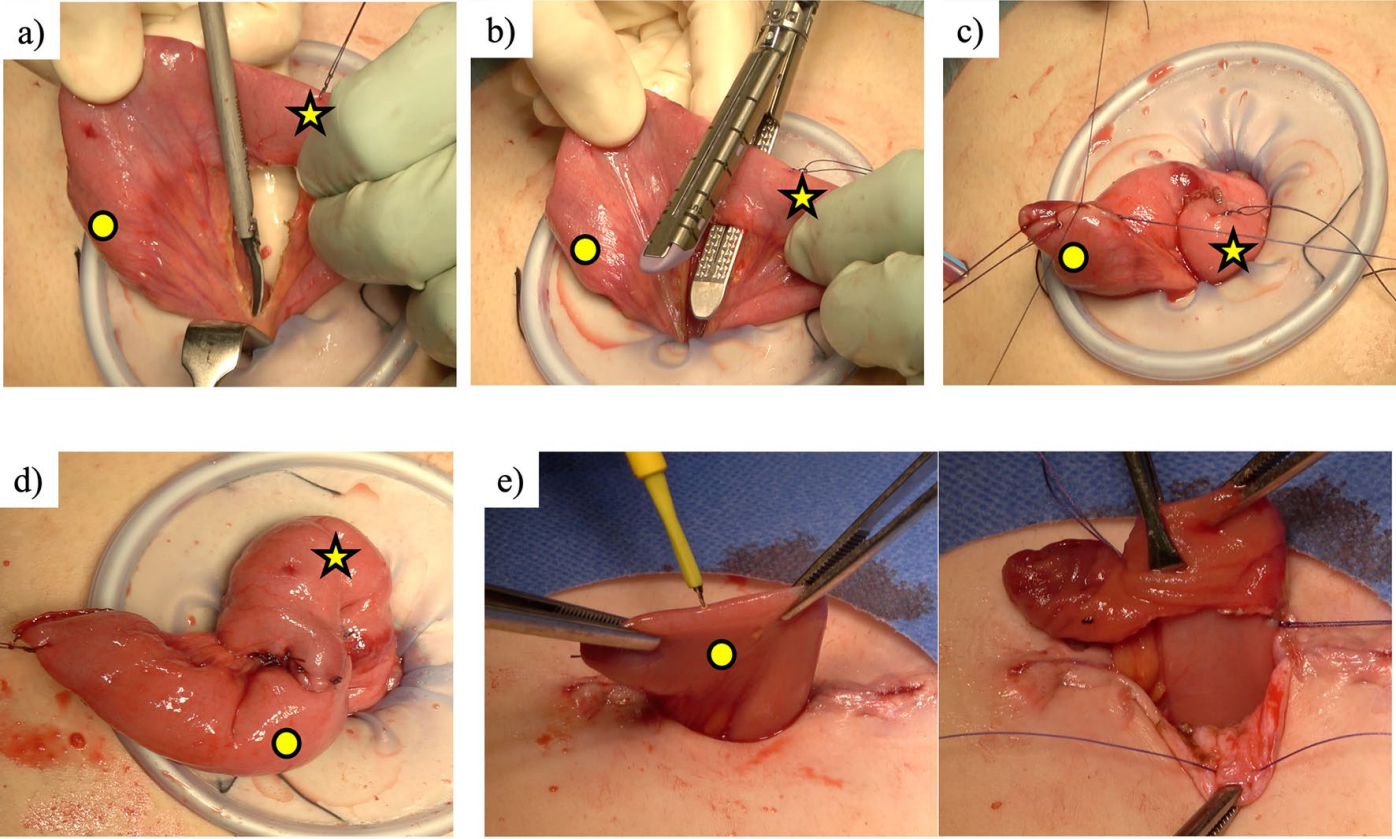
How to create loop end ileostomy at the umbilicus. The circle indicates the oral side, and the star indicates the anal side. **a** Partial division of the mesentery. **b** Transection of the intestinal tract. **c** Closure of the anal stump. **d** Add the fixation of the intestine for the next surgery for stoma closure. **e** Open at the top position

Regarding stoma creation, the LI was fashioned without rotation in the vertical orientation. In cases of EI, partial division of the mesentery with resection of marginal vessels was performed. The intestinal tract was then transected, followed by closure of the anal stump, fixation of the intestine for the next surgery of stoma closure, and opening of the intestinal lumen in the top position (Fig. 4).

### Definition of stoma outlet obstruction and high-output syndrome

These definitions have been previously described [5]. Briefly, SOO was defined as SBO that showed symptoms of intestinal obstruction, with caliber changes near the ileostomy detected by computed tomography, or which recovered with tube insertion from the stoma. High-output syndrome (HOS) was defined as stoma drainage of 2 L/day for > 2 days or 1.5 L/day for > 3 days.

### Statistical analysis

Statistical analyses were performed using JMP 16.2.0 (SAS Institute Inc., Cary, NC, USA). Welch’s t-test, Pearson’s Χ^2^ test, or Fisher’s exact test were used to compare patient and clinical characteristics. Data are expressed as the mean and standard deviation (SD) or the number of patients and proportion (%). To adjust for confounding by unbalanced covariates, we used a multivariable logistic regression model to estimate the odds ratios (ORs) for the incidence of SOO according to the type of ileostomy, HOS, sex, surgical approach, abdominal wall thickness, height-adjusted distance between rSMA-bEAS, and body mass index (BMI). Measurements of abdominal wall thickness and height-adjusted rSMA-bEAS have been previously described [5]. Significance was assessed using a 95% confidence interval (CI). *p*values of < 0.05 were considered to indicate statistical significance.

## Results

In total, 127 consecutive patients underwent proctocolectomy during the study period. After excluding 21 patients who underwent 1-stage surgery, modified 2-stage surgery, permanent ileostomy, or multiple organ resection, as well as those with insufficient medical records, data from 106 patients who underwent diverting ileostomy were analyzed. Of these, 75 (70.8%) underwent LI and 31 (29.2%) underwent EI with RPC and IPAA (Fig. 3).

The patient characteristics are shown in Table 1. The two groups were comparable in terms of sex, age at surgery, body mass index (BMI), preoperative nutritional status, and preoperative CRP level. The surgical indications of the two groups did not differ to a statistically significant extent; however, more than half of the patients in the EI group had UC-associated cancer/dysplasia. Although preoperative treatments were comparable with regard to biologics and immunomodulator use, preoperative steroid use was significantly higher in LI patients (65.3%) than in EI patients (38.7%; *p* = 0.0116). We also examined previously reported anatomical risk factors for SOO [2, 3, 5, 9]. The two groups did not differ in abdominal wall thickness, rSMA-bEAS, or height-adjusted rSMA-bEAS.

**Table 1:** Patient characteristics of the loop and loop end ileostomy groups

	Loop ileostomy (*n* = 75)	Loop end ileostomy (*n* = 31)	*P* value
Sex			
Male	50 (66.6)	21 (67.7)	0.9147
Female	25 (33.3)	10 (32.3)	
Age at surgery, years	49.0 ± 16.3	54.0 ± 18.2	0.5405
BMI, kg/m^2^	21.3 ± 3.9	21.1 ± 4.5	0.5499
Height, cm	166.1 ± 9.5	165.7 ± 7.7	0.8598
Body weight, kg	58.1 ± 13.5	59.6 ± 13.5	0.7649
Nutritional status (Onodera PNI)			
> 40	52 (69.3)	23 (74.2)	0.6168
≤ 40	23 (30.7)	8 (25.8)	
Preoperative CRP (mg/dl)	0.25 ± 3.76	0.11 ± 1.73	0.1227
Surgical indication			
UC (Cancer/ Dysplasia)	28 (37.3)	16 (51.6)	0.5488
UC (Severe/ Fulminant)	21 (28.0)	6 (19.4)	
UC (Refractory)	22 (29.3)	7 (22.6)	
FAP	4 (5.3)	2 (6.5)	
Preoperative medication*			
Biologics	30 (40.0)	11 (35.5)	0.6411
Steroid	49 (65.3)	12 (38.7)	0.0116
Immunomodulator	37 (49.3)	11 (35.5)	0.1926
Anatomical factors			
Thickness of abdominal wall, mm	22.8 ± 9.9	21.5 ± 10.2	0.5508
rSMA-bEAS, mm	304.8 ± 24.9	310.0 ± 20.9	0.4203
Height adjusted rSMA-bEAS, mm/cm	1.83 ± 0.11	1.88 ± 0.13	0.1349

Data are presented as n (%) or mean ± SD

*BMI* body mass index, *PNI* prognostic nutritional index, *CRP* C-reactive protein, *UC* ulcerative colitis, *FAP* familial adenomatous polyposis, *rSMA*, the root of superior mesenteric artery, *bEAS* the bottom of the external anal sphincter

*including duplicates

Surgery-related factors were evaluated (Table 2). There were no differences between the two groups in terms of operative time or blood loss. The proportions of surgical approaches were comparable between the two groups (*p* = 0.6283), and > 90% of surgeries in each group were performed using a laparoscopic approach. The two groups did not differ in terms of surgical strategy, anastomosis, or stoma site. Regarding postoperative factors, the incidence of intra-abdominal abscess and HOS were also comparable between the two groups (*p* = 0.9677 and *p* = 0.6162, respectively).

**Table 2:** Surgery-related factors and postoperative factors of the loop and loop end ileosotmy groups

	Loop ileostomy (*n* = 75)	Loop end ileostomy (*n* = 31)	*P* value
Surgery-related factors			
Operation time, min	398.0 ± 152.1	426.0 ± 202.3	0.1024
Blood loss, ml	220.0 ± 445.9	100.0 ± 316.0	0.3136
Surgical approach			
Open (Including conversion)	7 (9.3)	2 (6.5)	0.6283
Lap (Including Robot)	68 (90.7)	29 (94.5)	
Strategy			
2-stage surgery (RPC)	55 (73.3)	26 (83.9)	0.245
3-stage surgery (STC + RR)	20 (26.7)	5 (16.1)	
Anastomosis (IPAA)			
Hand-sewn	38 (50.7)	18 (58.1)	0.4877
Stapled	37 (49.3)	13 (41.9)	
Site of stoma construction			
Umbilicus	42 (56.0)	15 (48.4)	0.4711
Right abdomen	30 (40.0)	13 (41.9)	
Left abdomen	3 (4.0)	3 (9.7)	
Postoperative factors			
Intra-abdominal abscess	5 (6.6)	2 (6.5)	0.9677
High output syndrome	33 (44.0)	12 (38.7)	0.6162
Stoma outlet obstruction	20 (26.7)	3 (9.7)	0.0536

Data are presented as n (%) or mean ± SD

*SOO* outlet obstruction, *IPAA* ileal pouch-anal anastomosis, *RPC* restorative proctocolectomy, *STC* subtotal colectomy, *RR* residual rectal resection

The incidence rate of SOO in the EI group was 9.7% (*n* = 3), which was markedly lower than that in the LI group (26.7%, *n* = 20) (*p* = 0.0536). The severity of SOO was assessed using the Clavien–Dindo classification. In the LI group, 13 patients required ileostomy tube drainage with intravenous feeding (grade II), 5 patients required the insertion of an ileus tube (grade IIIa), and 2 patients required early stoma closure to manage SOO (grade IIIb). In contrast, in the EI group, 2 patients had grade II SOO and 1 had grade IIIa SOO. No patients in the EI group required early stoma closure.

We performed a multivariate logistic regression analysis using the total cohort to assess which factors were independently associated with SOO (Table 3). After adjusting for the stoma creation method, sex, BMI, anatomical factors, surgical approach, stoma site, and HOS, the EI group was found to have a significantly lower risk of SOO than the LI group (OR 0.18, 95% CI 0.03–0.92; *p* = 0.0399). HOS was a risk factor for developing SOO (OR 4.46, 95% CI 1.31–15.17; *p* = 0.0165) when other factors were assessed, and height-adjusted rSMA-bEAS was associated with a significantly higher incidence of SOO (OR 1.04, 95% CI 1.00–1.08; *p* = 0.0265).

**Table 3:** Multivariate logistic regression analysis

	SOO	Non-SOO	Univariate		Martivariate	
			Odds ratio (95% CI)	*P* value	Odds ratio (95% CI)	*P* value
Loop end ileostomy	3 (9.68)	28 (90.32)	0.29 (0.08–1.08)	0.0646	0.18 (0.03–0.92)	0.0399
Loop ileostomy	20 (26.67)	55 (73.33)	Reference		Reference	
High output syndrome yes	18 (40.00)	27 (60.00)	7.47 (2.50–22.25)	< 0.0001	4.46 (1.31–15.17)	0.0165
High output syndrome no	5 (8.20)	56 (91.80)	Reference		Reference	
Female	3 (8.57)	32 (91.43)	0.24 (0.07–0.87)	0.0299	0.68 (0.13–3.55)	0.65
Male	20 (28.17)	51 (71.83)	Reference		Reference	
Lap	22 (22.68)	75 (77.32)	2.35 (0.05–3.59)	0.433	3.07 (0.26–36.36)	0.3747
Open	1 (11.11)	8 (88.89)	Reference		Reference	
Thickness of abdominal wall (mm)	20.83 ± 10.84	23.26 ± 9.61	0.97 (0.92–1.03)	0.3165	0.95 (0.88–1.03)	0.2067
Height adjusted rSMA-bEAS (mm/cm)	318.17 ± 22.64	299.92 ± 22.36	1.04 (1.01–1.07)	0.0021	1.04 (1.00–108)	0.0265
BMI (kg/m^2)	21.16 ± 3.49	21.51 ± 4.20	0.98 (0.87–1.10)	0.7064	1.01 (0.84–1.20)	0.9362

Data are presented as n (%) or mean ± SD

*SOO* outlet obstruction, *BMI* body mass index, *Lap* laparoscopic surgery, *rSMA* the root of superior mesenteric artery, *bEAS* bottom of the external anal sphincter

## Discussion

Diverting LI for severe UC were first reported in 1913, and various stoma-related complications have been reported to date [10]. In particular, SOO prolongs the hospital stay and can lead to serious complications such as perforation. Therefore, in cases of repeated SOO, early stoma closure or re-creation of the stoma must be considered after evaluating the status of the anastomotic site. SOO rarely occurs within 1 week after surgery and mostly occurs after oral intake becomes sufficient [11]. In this study, all SOOs performed within 30 days of surgery were included. In 2021, diverting EI was introduced for all patients to reduce mesenteric tension, and this study revealed that EI significantly prevented SOO relative to LI.

Enterostomal therapists found LI more difficult to manage than EI, with more severe skin complications in LI [12, 13]. Effective management requires a 1.5-cm ileostomy protrusion, which is challenging with LI in IPAA cases but easily achieved with EI. Nonetheless, earlier investigations have demonstrated that the primary benefit of LI over EI for stoma closure is a reduced length of surgery [1]. Therefore, to facilitate anastomosis during stoma closure, we applied anti-adhesive agents and performed fixation of the intestine in the preceding operation (Fig. 4).

IPAA without a diverting stoma is performed when the status of anastomosis is quite good, but a large-scale study reported that approximately 75% of patients have a diverting ileostomy and 1-stage procedures are not common [2]. The ECCO guidelines suggest that modified 2-stage IPAA may have fewer complications and a shorter length of stay than 3-stage or 2-stage IPAA in expert centers [14]. However, previous studies have shown that the IPAA leakage rate is not low (approximately 10%) using a modified 2-stage approach [15]. In the absence of a diverting stoma, the pouch function and fertility are greatly affected by the occurrence of anastomotic leakage [16]; thus, selection requires careful consideration. Moreover, > 90% of patients in this study underwent diverting ileostomies with IPAA.

Previously reported risk factors for SOO include laparoscopic surgery, loop ileostomy, male sex, low BMI, rectoabdominal wall thickness at the stoma site, HOS, distance from the pouch to the LI (< 30 cm), and a long distance from the rSMA to the bEAS [2, 3, 5, 9, 17, 18]. Our study also revealed that a long distance from the rSMA to the bEAS and HOS was independently associated with a high risk factor of SOO. However, laparoscopic surgery was not a significant risk factor, because only a few patients underwent open surgery. Regarding sex, a significant difference was identified in the univariate analysis, but not in the multivariate analysis. Multiple studies have indicated ways to prevent SOO, such as making vertical fascia incisions instead of cruciate incisions, creating adequate space in the abdominal wall tunnel, and positioning the proximal side of the elevated intestinal tract in the 3 o’clock direction to minimize twisting of the mesentery [4, 5, 11]. In our study, EI was found to be an independent preventive factor for SOO.

IPAA requires pulling the ileal pouch into the pelvis, and previous studies have shown that 1.8% of patients could not undergo IPAA due to technical difficulties such as severe obesity, a large volume of mesentery, or ischemia of the pouch with mesenteric distraction [19, 20]. Although mesenteric panniculitis and sclerosing mesenteritis are both associated with thickened mesentery, there is no established consensus on the impact of steroid use in relation to these conditions. In this study, the lower frequency of preoperative steroid use in the EI group was likely due to the emergence of new treatment options (beyond steroids) over time. In terms of SOO, we considered the effect of steroid use to be minimal. The anastomotic site experienced pulling tension due to extension of the pouch mesentery [21]. Therefore, surgeons have implemented several measures to improve this, such as sufficient mobilization of the small intestine, division of the mesenteric serosa, vascular dissection, and manual guidance of the pouch [6–8, 20]. After introducing EI, there were no cases in which IPAA could not be performed. Although LI is easier and preferable without tension of the ileal mesentery, tension frequently exists in IPAA cases, unlike in rectal cancer cases. It seems reasonable to consider EI as an option if there is tension in the ileal mesentery and anastomosis remains difficult despite various countermeasures or if the stoma height cannot be maintained, as this would increase the risk of SOO.

The present study was associated with several limitations. First, all data were obtained from Japanese patients; therefore, the applicability of the results to other nationalities remains unclear. Second, the study was conducted using data obtained from a single center and was a retrospective observational study with a small sample size. A larger, long-term, prospective study is required to validate our findings.

In conclusion, this study demonstrates that EI can reduce SOO after RPC and IPAA. In addition, reduction of mesenteric tension using EI might be a solution for cases in which anastomosis is difficult.

## Data Availability

The data used in this study will be shared upon reasonable request by the corresponding author.
